# On-sensor binarized CNN inference with dynamic model swapping in pixel processor arrays

**DOI:** 10.3389/fnins.2022.909448

**Published:** 2022-08-15

**Authors:** Yanan Liu, Laurie Bose, Rui Fan, Piotr Dudek, Walterio Mayol-Cuevas

**Affiliations:** ^1^Bristol Robotics Laboratory, Faculty of Engineering, University of Bristol, Bristol, United Kingdom; ^2^School of Microelectronics, Shanghai University, Shanghai, China; ^3^Department of Control Science and Engineering, College of Electronics and Information Engineering, Tongji University, Shanghai, China; ^4^School of Electrical and Electronic Engineering, University of Manchester, Manchester, United Kingdom; ^5^Amazon.com, Seattle, WA, United States

**Keywords:** on-sensor computing, SCAMP vision system, pixel processor array, embedded computer vision, convolutional neural network

## Abstract

Many types of Convolutional Neural Network (CNN) models and training methods have been proposed in recent years aiming to provide efficiency for embedded and edge devices with limited computation and memory resources. The wide variety of architectures makes this a complex task that has to balance generality with efficiency. Among the most interesting camera-sensor architectures are Pixel Processor Arrays (PPAs). This study presents two methods that are useful for embedded CNNs in general but particularly suitable for PPAs. The first is for training purely binarized CNNs, the second is for deploying larger models with a model swapping paradigm that loads model components dynamically. Specifically, this study trains and implements networks with batch normalization and adaptive threshold for binary activations. Then, we convert batch normalization and binary activations into a bias matrix which can be parallelly implemented by an add/sub operation. For dynamic model swapping, we propose to decompose applications that are beyond the capacity of a PPA into sub-tasks that can be solved by tree networks that can be loaded dynamically as needed. We demonstrate our approaches to various tasks including classification, localization, and coarse segmentation on a highly resource constrained PPA sensor-processor.

## 1. Introduction

**Figure 1:**
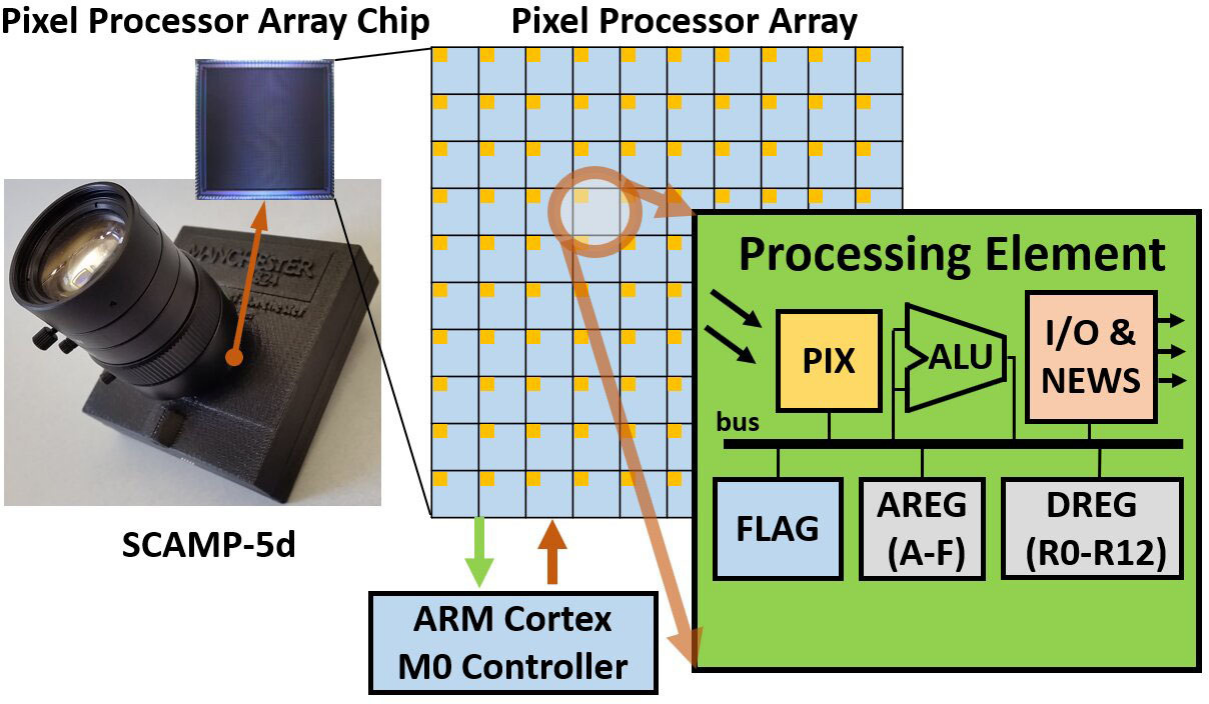
SCAMP-5d vision system and the pixel processor array (PPA). SCAMP-5d consists of PPA with 256×256 Processing Elements (PE) and ARM Micro-controller where parallel image processing is conducted on PPA by directly operating on an analog signal (electric current from PIX, which is proportional to the light intensity) within Analog Registers (AREGs) and bit operation within Digital Registers (DREGs).

Sensing, storage, and processing integration on a single chip are appealing for embedded real-time Convolutional Neural Network (CNN) inference. With an all-in-sensor scheme, a device can perceive the environment and generate useful results efficiently with reduced energy consumption, latency, and data bandwidth (Bose et al., 2017, 2019, 2020; Zhou and Chai, 2020). Pixel Processor Arrays (PPAs) (Figure 1) (Carey et al., 2013) are emerging programmable massively parallel vision sensors that integrate imaging, storage, and computation on the focal plane. Considering the distributed Processor Elements (PEs, Figure 1) of PPA, the neural network inference needs to be carefully implemented to fully take advantage of the sensor's parallel processing performance. However, full-precision neural networks usually require large amounts of memory space for weights and temporal results such as activations. Furthermore, floating-point computation, which is possible with modern CPU/GPUs, does not suit current on-sensor computing devices. With these challenges in mind, this study designs and trains purely binarized convolutional neural networks (CNNs) for PPA's hardware architecture and proposes new methods to deploy these proposed CNNs on the PPA across image classification, object localization, and coarse segmentation tasks with different neural network architectures.

For real-time execution of a deep neural network with millions of floating-point parameters, a powerful CPU/GPU is often required, which is incompatible with embedded visual sensors, such as SCAMP. As a result, we partition reasonably complicated tasks into smaller ones that may be executed within PPA's storage and register resource limits. More precisely, neural network models can be regularly uploaded into registers for computing based on the outcomes of the previous inference. In this case, only one neural network or segment of a network is performed at one time, while the rest of the models are saved in flash memory and can be dynamically uploaded into registers when needed. Specifically, we send input images through a sequence of networks, at each stage the output of the last network is used to determine which network is used next, effectively allowing a more complex task to be performed by a series of simple networks. We apply this scheme to classification, object localization, and segmentation tasks. In the experiments, we implement the proposed CNN tree architecture on the sensor and demonstrate it with: real-time 37-class English letter classification, objects' localization, and coarse image segmentation. The major contributions of this study can be summarized as follows:

(1) We train purely binarized CNNs (binary weights and activations) and implement them on the PPA for the first time. This approach of binary activation alleviates the accumulation of analog computing errors and value saturation after each layer, thus, enabling deeper neural networks on the sensor. With the binary neuron activations as inputs, the linear layers can be implemented by simply counting the number of bits (Liu et al., 2020a).
(2) We propose a dynamic-swapping CNN architecture where multiple neural networks can be composed for more sophisticated inference tasks by dynamically uploading neural network models when needed.
(3) We present the first implementation of a Fully Convolutional Network (FCN) architecture for PPAs. Our approach uses group convolutional layers (Wang et al., 2019), and stores hundreds of convolutional filter weights upon the focal plane of the PPA. Unlike earlier study, we apply batch normalization during training and utilize this to learn bias parameters to be applied during inference on the PPA device.

## 2. Related study

Pixel Processor Arrays combine sensing, processing, and memory. This helps to reduce the key factors on embedded systems such as latency, energy consumption, and bandwidth, as data movements are optimized and redundant information is eliminated close to its source. The SCAMP-5d vision system (Dudek, 2004; Carey et al., 2013) is a general-purpose programmable massively parallel PPA vision system that has been recently demonstrated in robotic tasks (Greatwood et al., 2017, 2018; McConville et al., 2020; Fan et al., 2021; Liu et al., 2021a) and computer vision (Bose et al., 2017; Chen et al., 2017; Martel et al., 2020). Figure 1, illustrates how the SCAMP's Processing Element (PE) uses a photosensor to convert light into an analog signal which is then directly processed in the adjacent arithmetic logic unit (ALU) and analog (AREG) and binary (DREG) registers. In contrast to current hardware design structures of computer vision systems, the PPA eliminates the need for Analog/Digital Conversion (ADC) after sensing and directly operates on analog electric currents, accelerating the signal processing speed and in the mean time, avoiding the bottleneck of ADC and data transmission process. Note, however, that noise can be introduced when performing arithmetic operations or temporal information storage on AREG (Zhou and Chai, 2020).

First examples of CNN implementation and inference within PPAs were demonstrated by Bose et al. (2019) where a CNN with a single convolutional layer and a single fully-connected layer is implemented upon the PPA array and its controller chip, respectively. Their study performs 16-bit image convolution operations using 4×4 DREG “Super Pixel” blocks and demonstrates live digit classification based on the MNIST dataset at a speed around of 200 FPS. In their study, the ternary –1, 0, 1 kernel filters are stored in the program memory, and are effectively encoded in the instructions/operations sent to the PPA array, performing convolutions in sequence. To fully take advantage of PPA's parallel computing characteristics and further improve the CNN inference efficiency, Bose et al. (2020), for the first time, proposed the idea of in-pixel weight storage, where the ternary weights are directly stored in DREG enabling a ×22 faster CNN inference (4,464 FPS) on the same digit recognition task with a similar network architecture by parallel image convolution and fully-connected layer. Based on these two studies, Liu et al. (2020a) proposed a high-speed lightweight neural network using BinaryConnect (Courbariaux et al., 2015) for multiple classification tasks with a new method of image convolution implementation using different high parameters (stride) across four different classification tasks including hand gesture recognition and plankton classification with frame rates ranging from 2,000 to 17,500 per second with different stride setups. Later, based on this network, a direct servo control using CNN results from Liu et al. (2021c) and a simulated robot tracking from a drone (Liu et al., 2021b) with on-sensor CNN computing results are exploited. Apart from this computer vision research, Chen et al. (2020) uses abstract features (mainly edges, corner points, and blobs) as inputs to a neural network for proximity estimation on a mobile robot platform, where feature extraction is performed on the PPA and a layer-recurrent network is carried out on the micro-controller. Other CNN-related study based on SCAMP can be seen from Wong et al. (2018, 2020) and Guillard (2019) where a CNN with a single convolutional layer of 3 kernel filters on the PPA and a single fully-connected layer on the M0 controller for digit recognition. However, their multiplication operation in convolution is approximated using combinations of additions and 1/2 divisions, where errors are introduced in theory and accumulated in practice which prevents it from a deeper network with many convolution filters. AUKE (Debrunner et al., 2019) is a useful tool to automatically generate convolution kernel codes on the PPA. Stow et al. (2021) is another compiler targeting the SCAMP-5 vision system developed by Stow et al. Furthermore, Martel et al. trained neural networks to learn pixel-wise exposures for HDR imaging and video compression (Martel et al., 2020).

Previous study by Bose et al. (2019), Bose et al. (2020), and Liu et al. (2020a), based on PPA for CNN-related research and implementation use binary weights and does not adopt batch norms in the training or inference process, the possible reasons are that to find a proper method to implement a binary CNN with the batch norm on PPA is challenging considering the PPA special hardware architecture and limited hardware resources and how much batch normalization can contribute to a shallow binary neural network on SCAMP remained unexploited. Based on the study of Bose et al. and Liu et al. above, our study proposed methods to train binarized CNNs across applications of letter recognition, object 2D localization, and coarse segmentation. Specifically, we use a series of techniques including batch normalization, group convolution, and activation function *tanh* to balance the network performance and deployment difficulties on the sensor.

## 3. Method

Neural network architectures for PPAs have to be carefully designed taking into account model size, architecture, and the feasibility of exploiting the PPA's parallel computation and on-sensor storage. This is essential due to the limited on-sensor resources compared to standard computer GPU/CPU hardware. This section attempts to find a balance between the neural network performance and its efficient implementation on current PPAs.

### 3.1. CNN with binary weights and activations

BinaryConnect (Courbariaux et al., 2015) trains neural network with binary weights during forward propagations. However, BinaryConnect is not a fully binary neural network with floating-point neuron activations. Both the CNN and FCN presented in this study are based on the Binarized CNN (Courbariaux et al., 2016) with both binary weights and neuron activations. Such binary values can be stored in 1-bit DREG and processed with bit-wise operations upon the PE array. Binarized CNN reduces the intermediate memory storage required for neuron activations and replaces most arithmetic operations with bit-wise operations. These qualities make such fully binarized neural networks highly suitable for PPAs.

During training, we employ a simple strategy to binarise the weights and activations. All the weights are efficiently binarized in a deterministic manner Equation 1.


(1) $$w_{b}=Sign(w_{r})=\{\begin{array}{ll} +1 & w_{r}>0,\\ -1 & otherwise \end{array}$$

where *w*_*r*_ is floating-point weights and *w*_*b*_ is the binary weights. In terms of activations, we train channel-wise adaptive threshold α to obtain more informative binary feature maps, inspired by the study by Liu et al. (2020b). In Equation 2,


(2) $$a_{b}=sign(a_{r}-\alpha )=\{\begin{array}{ll} +1 & a_{r}>\alpha ,\\ -1 & otherwise \end{array}$$

α is the trainable threshold for binarization of each channel, *a*_*r*_ is the full-precision activations and *a*_*b*_ is the binarized activations. The key to the back propagation is the gradient calculation and accumulation for Stochastic Gradient Descent (SGD). During the training process, using standard back-propagation and stochastic gradient descent, the gradients are calculated with the floating-point weights. The weights and activations are only binarized during the forward pass. In our study, the binarized CNN is trained on PC and the CNN inference process is implemented on the PPA of the SCAMP-5d vision system.

**Figure 2:**
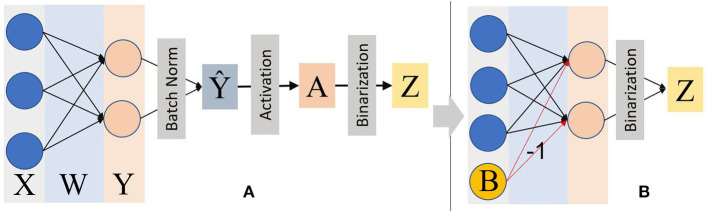
**(A)** The binarized CNN forward propagation with the batch norm and adaptive activation function before simplification. The activation function *tanh* is used considering its feature of sign invariance to inputs. **(B)** The simplified inference process on the PPA device by mathematically transforming batch norm, activation function into a “bias” ***B*** to be subtracted from ***Y***. With this method, the whole inference is not only significantly simplified but also transformed all the multiplication into addition/subtraction operations.

The training process for batch norm parameters can be seen by Ioffe and Szegedy (2015). From Equations 3 to 8, ϵ is used to avoid a zero denominator. The main scaling and shifting parameters γ and β for batch norm are learned during the training process. Then the batch norm can be applied to manipulate activations (Ioffe and Szegedy, 2015). In Figure 2, for a mini-batch *B* = {*x*_1_, *x*_2_, …, *x*_*n*_} and a single layer of Binarized CNN during the forward propagation process:


(3) $$\begin{array}{l} Y=\overset{n}{\underset{i=0}{\sum }}w_{i}x_{i} \end{array}$$

(4) $$\begin{array}{l} Ŷ=\gamma \frac{Y-\mu }{\sqrt{\sigma^{2}+\epsilon }}+\beta =\frac{\gamma }{\sqrt{\sigma^{2}+\epsilon }}\left(Y-\left(\mu -\frac{\sqrt{\sigma^{2}+\epsilon }}{\gamma }\beta \right)\right) \end{array}$$

Considering activation function *tanh* and positive scalar does not change the sign of inputs. Hence,


(5) $$\begin{array}{l} \begin{array}{c} Z=sign\left(A\right)=sign\left(tanh\left(Ŷ-\alpha \right)\right)=sign\left(Ŷ-\alpha \right)\\ \;=sign\left(Y-\left(\mu -\frac{\sqrt{\sigma^{2}+\epsilon }}{\gamma }\beta \right)-\alpha \right) \end{array} \end{array}$$

Hence:


(6) $$\begin{array}{l} Z=sign\left(Y-B\right) \end{array}$$

Where,


(7) $$\begin{array}{l} B=\mu +\alpha -\frac{\sqrt{\sigma^{2}+\epsilon }}{\gamma }\beta \end{array}$$

In Equation 7,


(8) $$\begin{array}{l} \sigma^{2}=\frac{1}{n}\overset{n}{\underset{i=1}{\sum }}{\left(x_{i}-\mu \right)}^{2},\mu =\frac{1}{n}\overset{n}{\underset{i=1}{\sum }}x_{i} \end{array}$$

β, γ, and α are all trainable parameters that can be obtained directly after training. Thus, the “Bias” ***B*** can be calculated using these trained parameters offline before implementing it on the PPA. During the inference process on the sensor, the batch norm, activation function, and learnable threshold are reduced to a bias term, as shown in Equation 7. Hence, the on-sensor inference process can be simplified as shown in Figure 2B.

### 3.2. Dynamic model swapping and CNN tree

Device constraints for embedding visual systems invite us to reflect on alternative ways in which architectures can be developed and deployed. Especially when considering massively parallel and low lag hardware such as the SCAMP-5 PPA. Of interest is to be able to deal with challenging and complex tasks which due to device constraints are not possible to directly port to embedded devices. One opportunity here is to partition larger models and tasks into sub modules and sub tasks that can then be deployed.

**Figure 3:**
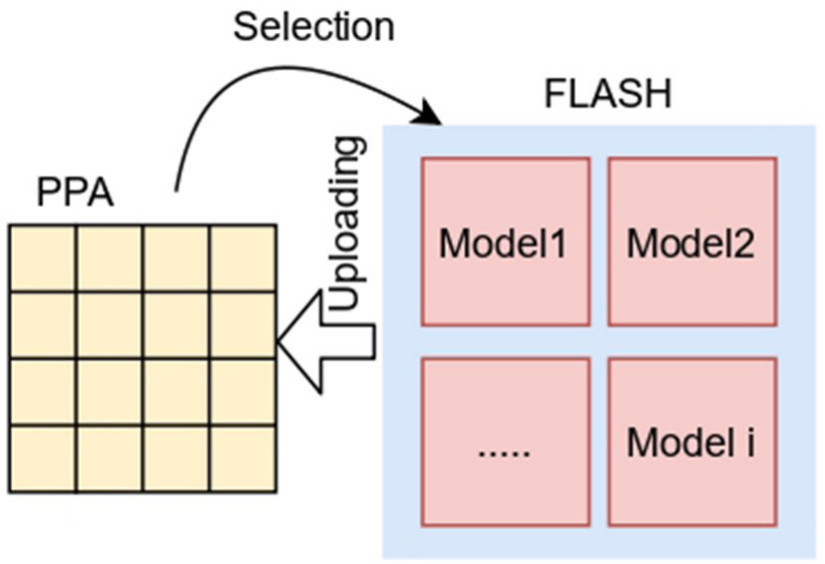
Neural network dynamic model swapping for the SCAMP.

Considering that the overall storage space in a SCAMP vision system (which includes a PPA as well as a microcontroller with its own RAM and flash memory) is much bigger than the one available in the PPA computing registers, it is possible to store several models in the flash memory and upload them dynamically in real time to the PPA registers for specific computation (Figure 3).

**Figure 4:**
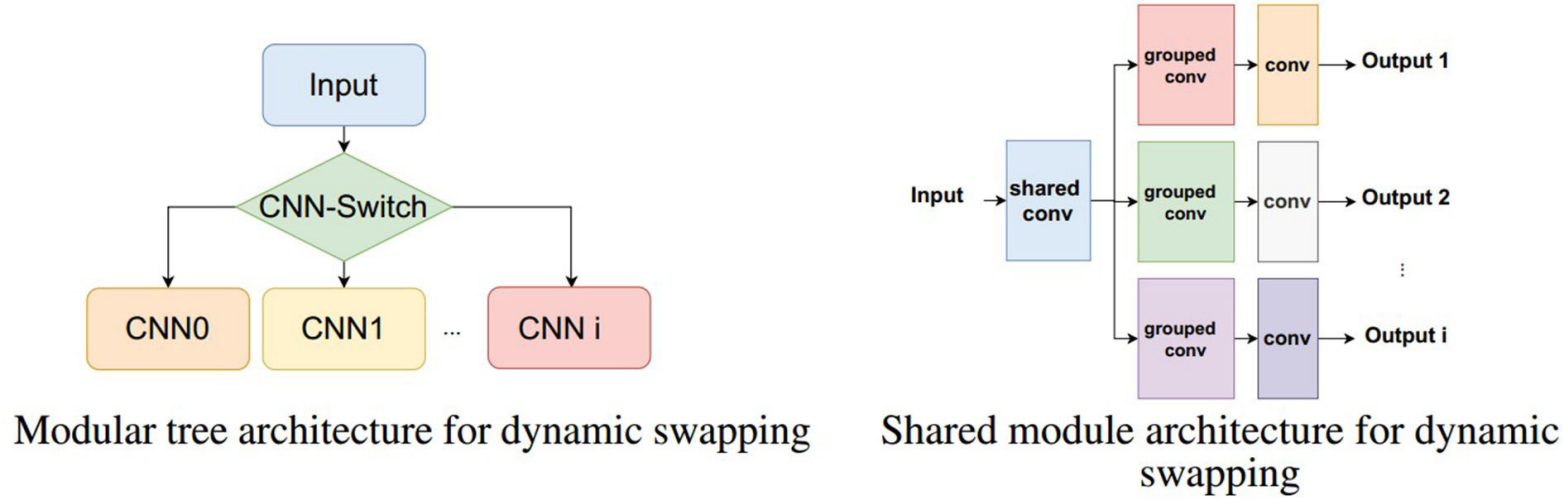
Two examples of dynamic swapping architectures. **(Left)** A CNN classification tree where each module is loaded dynamically as needed based on the switching CNN. **(Right)** A shared convolution module first processes the input and each distinct task use its own sub-modules that are loaded one at a time to produce the different outputs.

This allows designing new ways in which CNN and inference models, in general, can be deployed. For example, classification network trees with CNNs can be constructed. Specifically, there can be an input signal (image) that can first be assessed by a first switching network with reference to which downstream model should process it next. This, therefore, reduces the complexity of classification into binary or a few classes which can then be repeated in levels as necessary (Figure 4, Left).

Another alternative is to have shared processing models that are followed by task-specific sub models (Figure 4, Right). In this case, consider that a device is having to perform different tasks on the same input image, e.g., detecting objects and segmenting image regions. The first shared module could then be the first few convolutional layers of a model which use the entirety of the PPA. The result can then be followed by sub modules that are dynamically and in real time loaded to the PPA which implement grouped convolutions that are task-specific e.g., object detection and/or specific region segmentation. In this case, we trade overall speed for the ability to fit multiple tasks for which some portions are shared, removing redundant computation.

In this article, we explore initial implementations for dynamic swapping models for tree CNN architectures and shared convolutions.

## 4. CNN architecture on sensor

**Figure 5:**
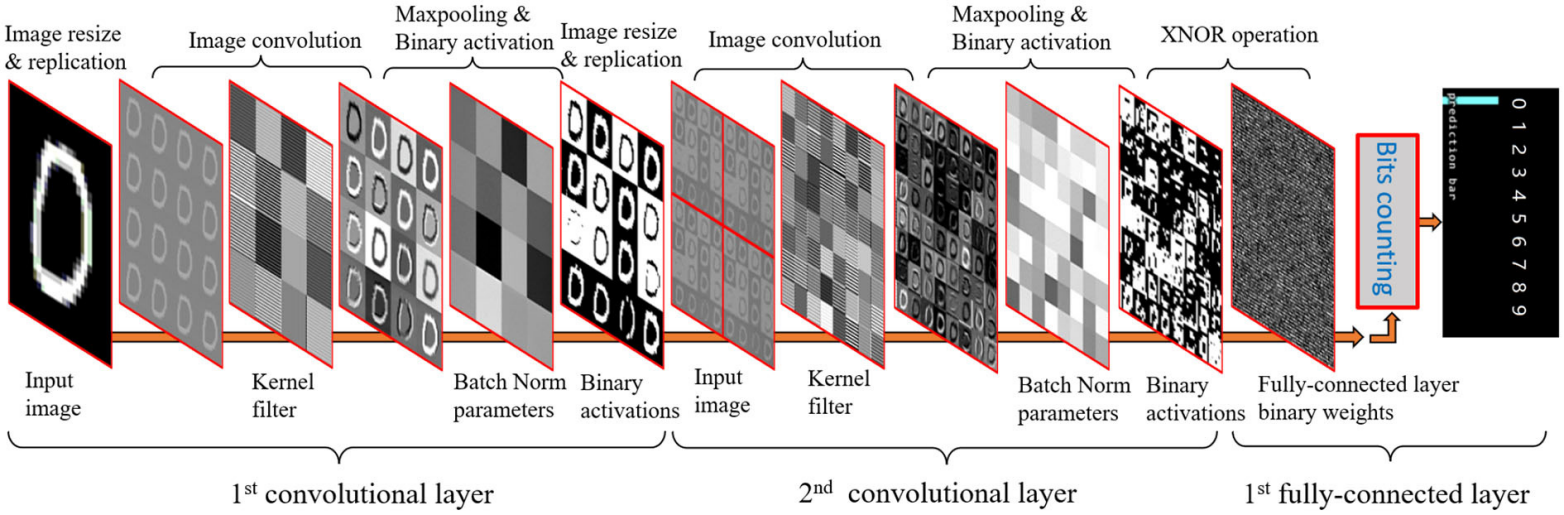
Convolutional neural network (CNN) inference process with multiple layers on the PPA by integrating image sensing, storage, and calculation using both DREG and AREG.

The overall binarized CNN architecture for sensing, storage, and computing can be seen in Figure 5. First for imaging, the photo detectors (PIX) within each PE convert light into analog signals which can be directly transferred and temporarily stored into AREG. The input image on AREG is then resized and replicated to fill the whole 256×256 PE for parallel processing purposes. With the binary convolutional weights stored in DREG, the image convolution can be performed using the replicated image and its associated weights. As illustrated in Section 3, the batch norm can be efficiently implemented by subtracting a matrix that is plotted in AREG. The binary activations can be obtained by binarizing the full-precision activations after being subtracted by the bias matrix. Then the binarized activations act as the inputs for the next convolutional layer using a similar process as the first layer. The fully-connected layer receives the binarized activations as an input; given binary weights, the final prediction can be created by conducting multiplications with the *XNOR* operation and then counting the bits for each label. The maximum number of bits indicates the final CNN inference result. The following sections detail the implementation method for each layer.

### 4.1. Convolutional layer

As shown in Figure 5, the image convolution can be executed in parallel on the PPA by “Multiplications”, shifting, and add/sub. First, the image information stored across the PE array is ‘multiplied’ by binary filter weights of –1,1 stored in another DREG. Then by shifting and adding horizontally 3 times and vertically for another 3 times, convolution results for the bottom right cell of each 4×4 PE block are generated. After repeating this process 16 times in a similar manner, an image convolution with stride 1 can be obtained. Image convolution with different strides can be implemented by different shifts (Liu et al., 2020a). Each 4×4 kernel filter is replicated across a 64×64 block (16 blocks in total), allowing multiple convolutions to be calculated in parallel. This study implements a CNN with two convolutional layers, the resolution of input images to PPA is 256×256 and after resizing and replication, 16 64×64 (16 images and each of them has a resolution of 64×64) images are inputs for the first convolutional layer followed by 2×2 max-pooling. For the second convolutional layer, a group convolution (Wang et al., 2019) with 16 groups and 4×4 max-pooling is utilized to simplify the calculation, reduce the memory requirement, and accelerate the network inference process. The input image for the second convolutional layer (shown in Figure 5) contains four identical images (128×128) in each of them there are 16 feature maps. After an image convolution with a group of 16, 64 feature maps are then generated simultaneously which act as inputs for the next CNN layer. With this method, there is no need to shift feature maps and add/subtract them into a new one as the normal convolution with a group of 1 does. For the second convolutional layer, hence calculation errors based on the analog signals can be reduced and the inference process can be accelerated with fewer register-based operations. More details about the kernel filter layouts and convolution implementation can be seen in Supplementary Figures S1, S2.

### 4.2. Fully-connected layer by bit counting

**Figure 6:**
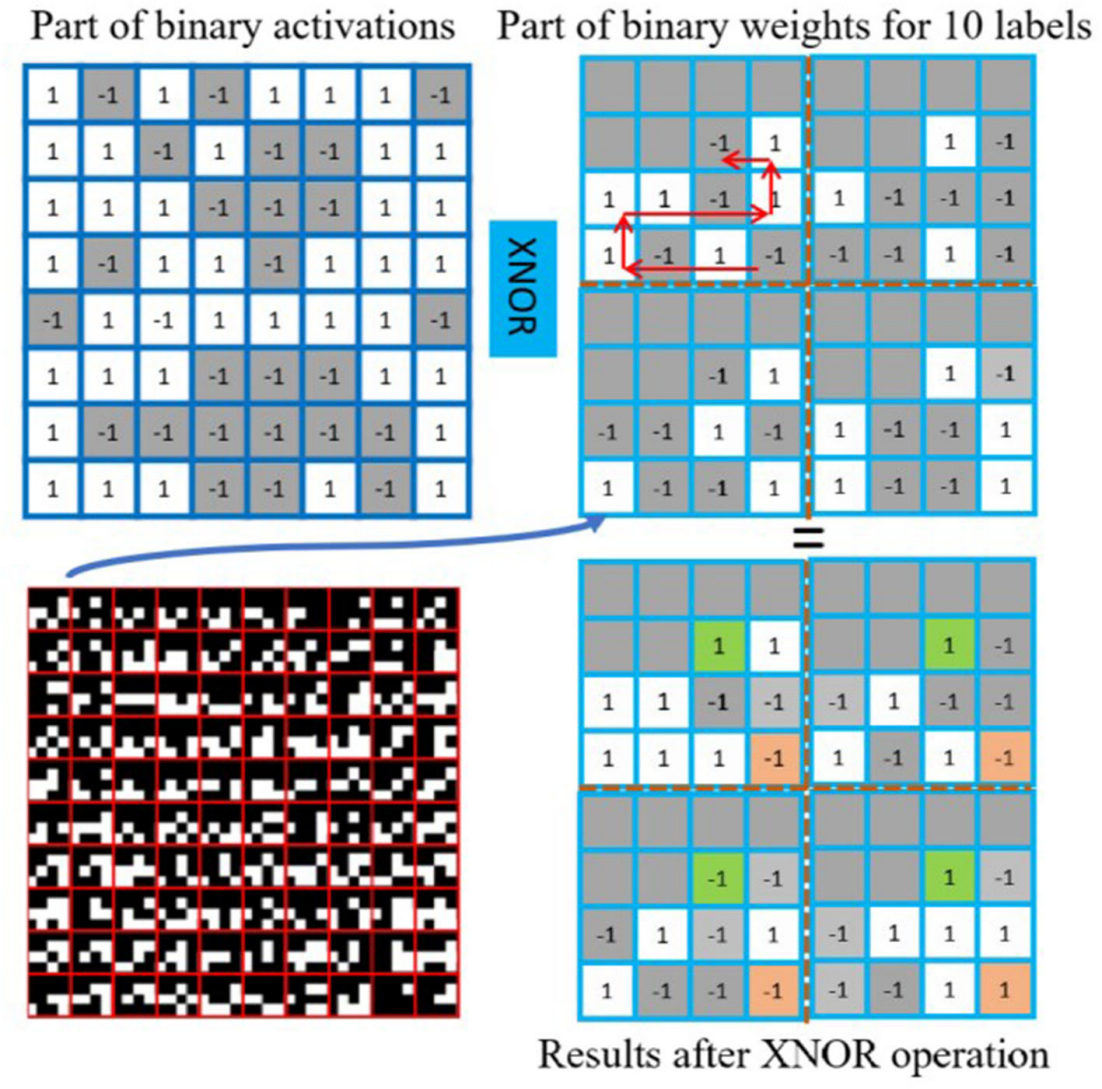
Fully-connected layer implementation. The fully-connected layer on PPA uses parallel XNOR operation and bit counting. The binary weights for labels are stored on DREG in a specific order within a 4×4 box. For example, the results for label 0 can be obtained by counting the sum of values in orange boxes (−1 − 1 − 1 + 1 = −2). The results for label 9 from green boxes (1 + 1 − 1 + 1 = 2).

As shown in Figure 6, the first step for the fully-connected layer is to multiply input binary feature maps with 1-bit weights. This is achieved by performing the *XNOR* operation between the given binary activations (Figure 6 top left) and weights (top right), which can be efficiently processed with the parallel bit operation on DREG. This figure shows the weights for 10 labels and they are pre-stored in a “snake” pattern in a 4×4 block on a DREG. Different from earlier study (Bose et al., 2019, 2020; Liu et al., 2020a) where the fully-connected layer is implemented mainly by using the built-in *scamp5_global_sum* function to estimate the summation of values in AREG, which is not accurate to fully represent the CNN outputs, in our study, the final CNN output can be obtained by activating each label's position shown from the bottom right in Figure 6 and counting the amount of positive and negative bits, which can be more accurate than approximation summation method. The pixel counting accuracy can be found in Supplementary Figure S3, which shows the similarity between a simulation on PC that can be regarded as ground truth and binarized CNN on the PPA.

### 4.3. Binary activation, batch norm, and max-pooling

This study uses *tanh* as the activation function for the convolutional layer because *tanh* does not change the sign of inputs. Hence, it could simplify the batch norm calculation on the PPA while maintaining a satisfactory accuracy as illustrated in Section IV. The *ReLU* is applied for the second fully-connected layer on the micro-controller to improve the overall performance of the CNN. In terms of the implementation of the activation function, the *tanh* activation function is transformed into batch norm weights as can be seen in Figures 2, 5. For a CNN with two convolutional layers, max-pooling with 2 and 4 is used respectively to cooperate with the parameter of group 16. With this combination, there is no need for multiple shifts and addition operations on AREG to get the final activations after the second convolutional layer as described in Bose et al. (2020). Hence, the second image convolution is simplified significantly while keeping a suitable accuracy.

### 4.4. Bit counting for the fully-connected layer

When calculating the neuron activations from the fully connected layer, it is necessary to count the number of set bits in a DREG. In this study, we make use of the “sandcastle summation” method (Supplementary Figure S4)^1^ (Bose et al., 2021). In brief, this method provides an efficient way to calculate an exact count of the number of set bits in a DREG. It achieves this by manipulating the DREG's content *via* efficient parallel operations, into a form where the number of set pixels can be easily determined. Specifically forming a stacked “sandcastle” of set pixels, after which the number of set pixels can be determined by calculating the area of this “sandcastle” stack. This approach is typically two orders of magnitude faster than a naive approach of counting set pixels individually.

## 5. FCN on sensor

**Figure 7:**
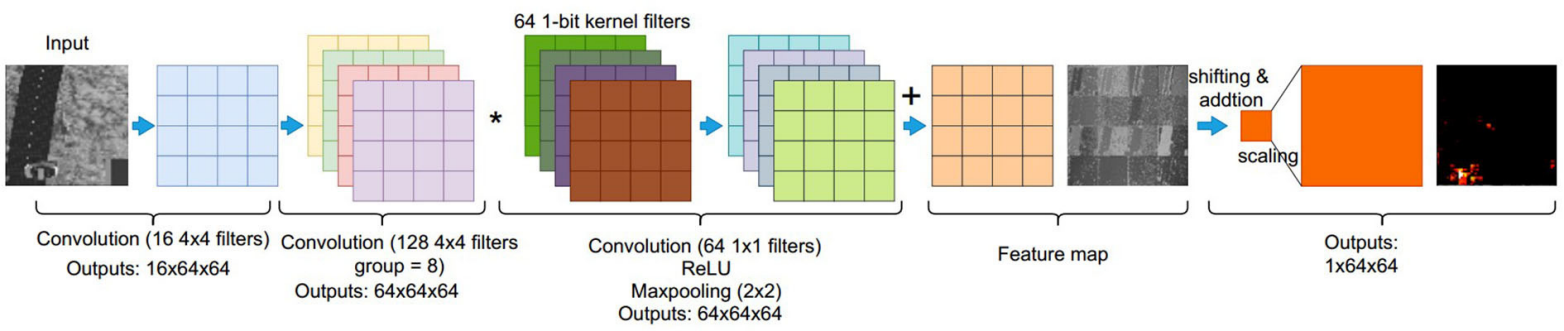
An overview of an on-sensor fully convolutional network (FCN) architecture and inference process using a PPA for heat map generation. A three-layer FCN architecture is used in our study. The first convolutional layer can be seen in Figure 5 in detail. In the second convolutional layer, 128 convolutional kernel filters are applied to the 16 input binary feature maps from the first layer, generating 64 feature maps with a convolution group setup of eight. The fusion of intermediate extracted features is implemented by addition within each group. The third layer uses binary filters with a size of 64 × 1 × 1, hence, the final feature maps can be obtained by “Multiplication” with bit operation based on DREG. The final heat map is generated by combining these input 64 feature maps by shifting and addition operations.

An FCN is a type of convolution neural networks that only performs convolution operations without using fully-connected layers, which provide pixel-level classification, targeting image segmentation (Long et al., 2015). This article proposes a 3-conv layer FCN that can be implemented on a PPA sensor. This study extends from previous CNN classifications, that were done using fully-connected output layers (Bose et al., 2020; Liu et al., 2020a). In this article, FCN is used for heat map generation by adding one convolutional layer with 128 filters and replacing the final fully-connected layer with a convolutional layer of kernel size 1×1. Figure 7 shows the overall FCN architecture with configurations for each layer. Figure 5 illustrates the first convolution layer, generating 16 binary feature maps. The second layer adopts group image convolution (Chollet, 2017) of 8 on the input 16 feature maps to make a trade off between convolution computation complexity and network performance, where each of 64 outputs is generated by adding two intermediate feature maps (**Figure 9**). These 64 binary feature maps from the second convolutional layer are stored in 4 DREG. The third layer then generates the final heat map representing the prediction probability distribution, taking these 64 binary feature maps and combining them within an AREG. Each binary feature map is multiplied by an associated weight of –1/1.

### 5.1. FCN deployment on the PPA

This study proposes a 3-conv layer FCN that can be fully parallelly implemented on the PPA. As can be seen from **Figure 9**, the first layer of FCN shares the same architecture as the CNN in Figure 5. Then, 16 binary feature maps are generated after image convolution, batch norm, and adaptive binarization in the first layer. To fully use the information from the given image and efficiently send outputs to the next layer, there is no max pooling applied in this layer. The second layer adopts group image convolution (Chollet, 2017) of 8 on the input 16 feature maps to make a trade off between convolution computation complexity and network performance, where each of 64 outputs is generated by adding two intermediate feature maps. These 64 binary feature maps are then stored in 4 DREG as can be seen from the outputs of the second convolutional layer. In the third layer, to generate the final heat map representing the position probability distribution, these 64 binary feature maps from the last layer need weighted by –1/1 and then summed up. First of all, after 1 × 1 convolution, these 64 feature maps stored in 4 DREG are relocated to 1 AREG after 2 × 2 max pooling. Then the summation of these extracted feature maps can be obtained by shifting and adding into one 64 × 64 heat map which represents the position distribution probability of the object. It is worth mentioning that for the second layer, we combine parallel and sequential implementation of the convolution, where for each iteration out of four in total, 16 feature maps are generated. Compared to our previous 2-layer network (Liu et al., 2020a) where 29,056 parameters are needed for a binary classification task, this 3-conv layer neural network has only 2,656 parameters which significantly alleviates the storage pressure for the embedded vision system, while in the meantime, generates more informative results such as the object 2D position and segmentation information within an image. The following section gives the implementation detail of the binarized FCN on the sensor.

**First Layer:** Sixteen binary filters are replicated to fill a DREG for parallel convolution purposes (Bose et al., 2020; Liu et al., 2020a). The image convolution on the PPA can be decomposed as ‘multiplications’, shifting, addition, and the convolution result are obtained by performing the shifting and addition process 16 times with a stride = 1. Then the pre-calculated bias *B* is input into 4×4 grids on an AREG and is subtracted from the feature map (Figure 5). Then the output binary image is obtained by binarizing the feature map after subtracting *B*. In this layer, *tanh* is used as the activation function. When implementing inference on the sensor, the *tanh* activation function is transformed into binarization with a sign function, with offset computed from batch norm parameters as can be seen from Equation 7.

**Figure 8:**
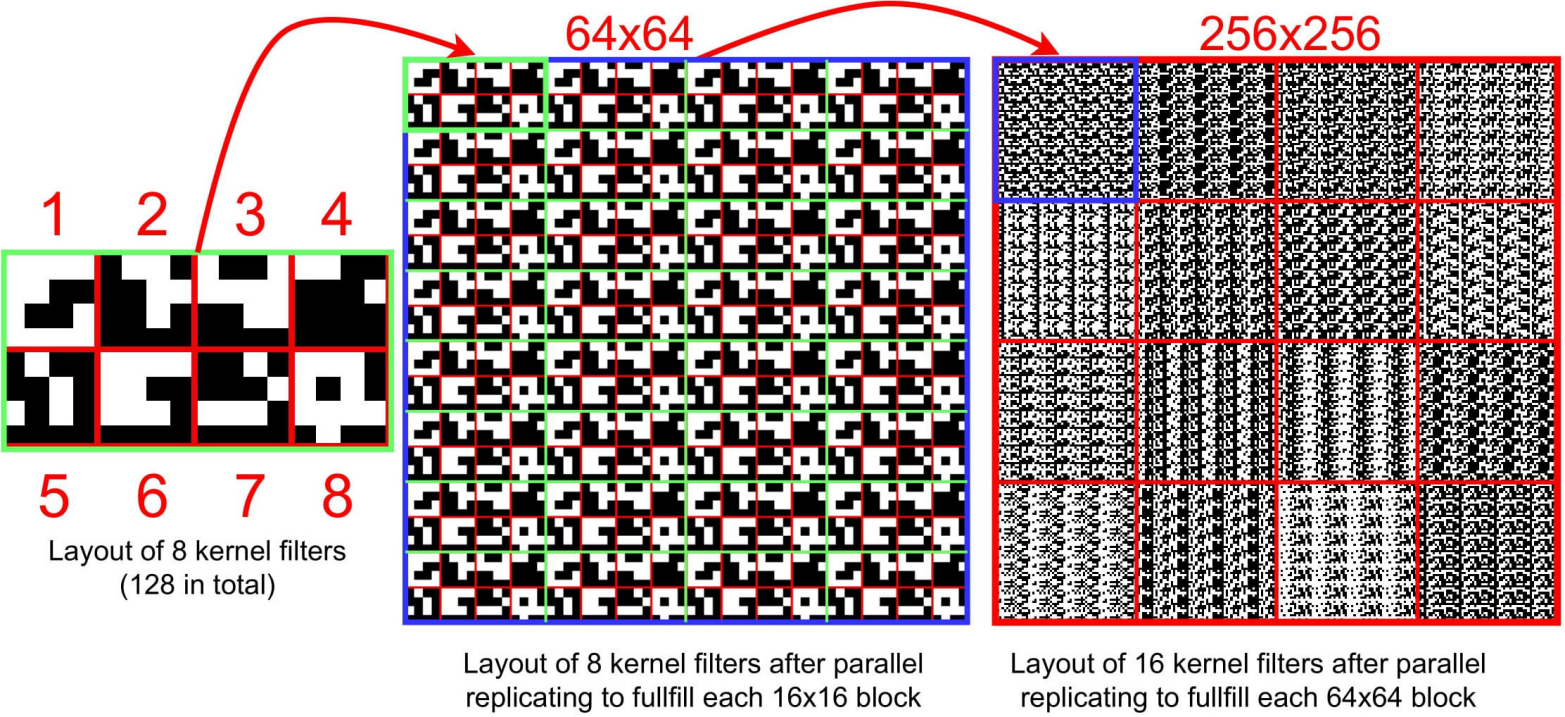
**(Left)** The layout of 8 filters in a block with a size 16×8. **(Middle)** The layout of 8 kernel filters in a 64×64 block. **(Right)** The layout of 128 kernel filters. Before performing a convolution operation (8 times in total) in the 2nd layer, each set (8 sets in total) of kernel filters is activated and then replicated to fill all 256×256 PEs. With this method, 128 filters can be stored within one DREG.

**Figure 9:**
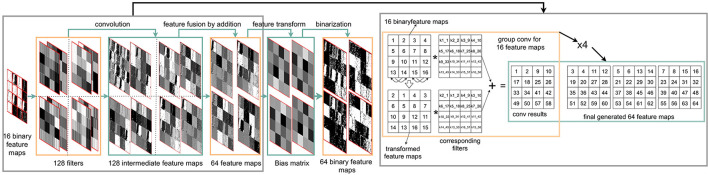
An overview of an on-sensor FCN inference process within the PPA for object localization. The first convolutional layer uses the same convolution module with CNN in Figure 5. As for the second convolutional layer, 128 convolutional kernel filters are applied on 16 input binary feature maps, generating 64 feature maps with a convolution group setup of eight. The fusion of intermediate extracted features is implemented by addition within each group. The third layer uses binary filters with a size of 64 × 1 × 1, hence the final feature maps can be obtained by ‘multiplication’ with bit operation based on DREG. The final heat map is generated by fusing these input 64 binary feature maps by only shifting and addition operations. More details on group convolution implementation can be seen in Supplementary Figure S5.

**Second Layer:** Group convolution is an advantageous approach for embedded devices due to the reduced number of parameters generated by group computing. The key to the second layer is to implement the group convolution with 16 feature maps as inputs and 64 feature maps as outputs. By dividing 16 input feature maps into eight groups, thus, there are 128 binary filters need to be stored on the sensor. The layout of filters directly affects inference efficiency. We design a storage structure for filters in the first (Supplementary Figure S1) and the second layer. Figure 8 illustrates the layout of these filters within one DREG. As can be seen, each time to perform a convolution, the corresponding kernel filters are activated in parallel, shifted, and replicated to fill each 64×64 block in 256×256 PEs. This filter storage structure can also extend to store more filters following a similar way to fill all PEs with 16 filters. In Figure 9, to implement a second convolution layer with 8 groups, the input 16 binary feature maps are first transformed by switching the position of adjacent maps. This is followed by convolution with associated 32 filters for these 32 feature maps. Then 16 gray-scale feature maps are obtained by adding each two of the 32 maps. By performing convolution for another 96 filters, 64 gray-scale feature maps can be derived. The bias matrix is subtracted and then after binarization, 64 binary feature maps are generated.

**Third Layer:** In this layer, as shown in Figures 7, 64 1-bit filters are input into a DREG, followed by ‘multiplication’ with the 1-bit feature maps from the previous layer. After 1×1 convolution, these 64 feature maps in 4 DREGs are relocated to 1 AREG after 2 × 2 maxpooling. The summation of these extracted features can be obtained by shifting and adding them into one 64 × 64 heat map (shown in Supplementary Figure S6). Unlike in the previous two layers, the activation function for this layer is ReLU to generate a gray-scale feature map as the final prediction result of the network.

## 6. Experiments

**Figure 10:**
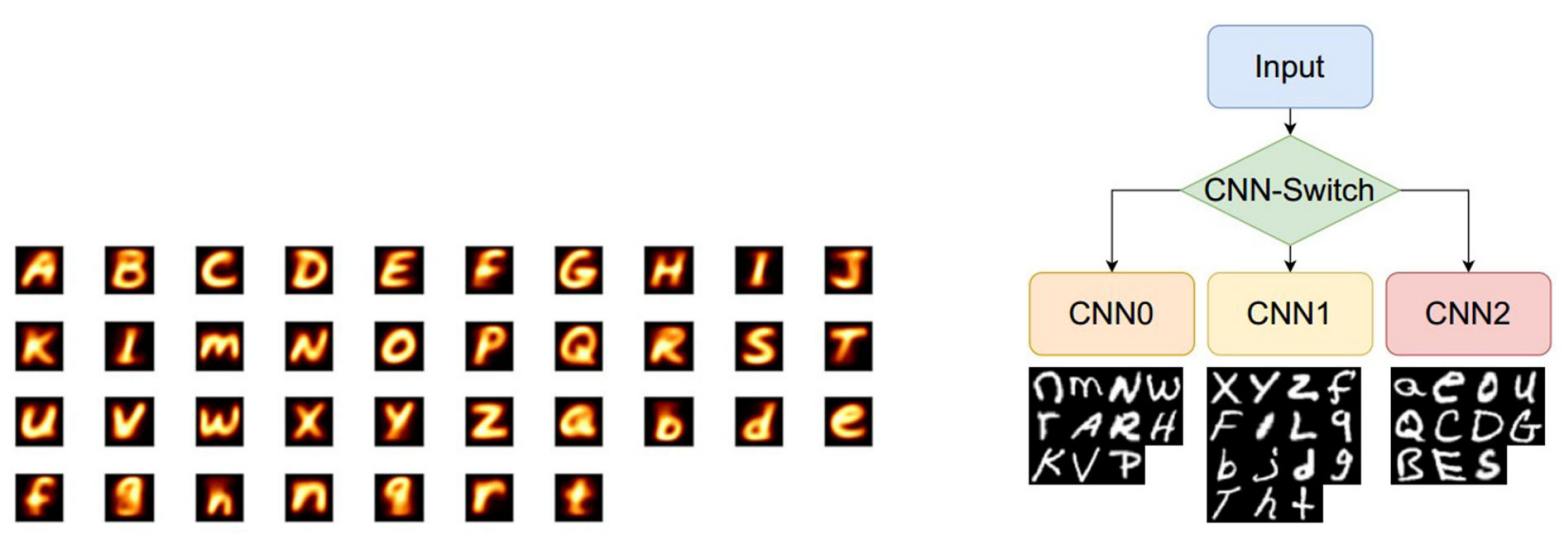
Letters from the EMNIST dataset and their three categories. **(Left)** The visualization of the average value for each letter from the EMNIST dataset. As can be seen, several classes share lots of similarities to some degree, such as *I and L, f and F, h and n, g and q*, and *Q and a* which makes this classification task challenging. **(Right)** CNN tree architecture using dynamic model swapping on SCAMP in which each CNN performs comparatively simpler tasks using the 4-layer CNN. These 4 categories using EMNIST merged classes.

This section demonstrates experiments, classification, localization, and segmentation, based on the proposed binarized CNN and FCN architecture, respectively. Figure 5 shows the design of a binarized CNN architecture as a node of the CNN tree for 37 English letters (Figure 10, Left) recognition. FCN architecture for object 2D localization, road, and grass coarse segmentation is validated in this section.

### 6.1. CNN tree on EMNIST for English letter classification

**Figure 11:**
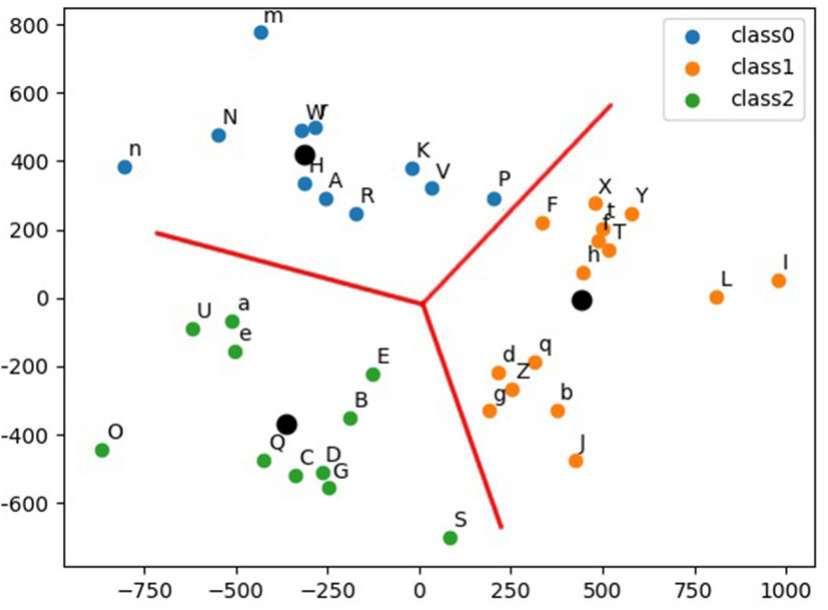
Thirty-seven English letters are clustered into 3 categories. Black dots represent the center of each category.

The EMNIST dataset (Cohen et al., 2017) is a set of handwritten characters extended from the MNIST (LeCun et al., 2010) dataset with the same image format. This study uses the merged class which contains 11 lower-case classes, 11 upper-case classes, and 15 mixed classes where some of letters are difficult to distinguish from upper case to lower case, such as *O, X*, and *C*. Hence, in total, there are 37 types of labels in the merged class. Considering the scarce hardware computing resources, especially the amount of DREG/AREG, to store weights, temporary activations, and perform convolution, this article proposes a CNN tree architecture consisting of 4 CNNs (Figure 10, Right), where the network can be swapped simply by loading associated weights from the flash memory to the DREG because all these four networks share an identical architecture. The overall 4 CNN structure is shown in Figure 10. The three categories of these 37 letters are obtained using k-means clustering *via* Principal Component Analysis (Ding and He, 2004) shown in Figure 11.

With the proposed CNN tree where each CNN uses 2 convolutional layers + 2 fully-connected layers, better accuracy of 86.74% is obtained compared to a single neural network with group 1 or 16 (Table 1). As can be seen from Figure 5, a third convolutional layer is challenging to extend to improve the CNN performance because the size of the feature map is 8×8 after two max-pooling (2 and 4) which is too tiny to add another max-pooling for the third convolutional layer. An alternative would be more fully-connected layers but it is also limited by the hardware resources. To improve the overall classification accuracy, a CNN tree with 4 CNNs (Figure 10) is proposed, where the basic idea is to use a combination of four 4-layer neural networks uploaded into SCAMP in sequence to get a closer accuracy with a deeper neural network that exceeds the SCAMP hardware storage/computation capacity. It might be challenging to store all the weights for a deeper neural network directly on a sensor-processor chip, but the parameters in each branch can be uploaded into the system from external memory, according to the last inference result, which alleviates the storage pressure and at the same time, obtains a better accuracy than a single neural network. In terms of the CNN tree training, four CNNs are trained separately with the same neural network structure. The accuracy for each CNN can be seen in Table 1. In addition, Supplementary Figure S10 shows the binary training process and comparison between a single network and a CNN tree. Note that CNN-1 suffers from a poorer performance compared to its counterparts resulting from not only the number of classes (15 vs. 11) but also the number of similar classes, such as *F* and *f*, *L* and *I*, and *g* and *q* shown in Figure 10, Left.

**Table 1:** Convolutional neural network (CNN) classification accuracy among different CNNs.

**CNNs**	**Accuracy**	**Parameter amounts**
CNN switch	95.55%	132,873
CNN 0	95.27%	133,153
CNN 1	84.74%	133,293
CNN 2	94.44%	133,153
Overall	**86.74**%	–
Single 4-layer CNN	**84.20**%	134,063
Single 4-layer CNN	**84.55**%	149,423
Group =1 (2nd conv)		

*The bold value indicates the classification accuracy*.

**Figure 12:**
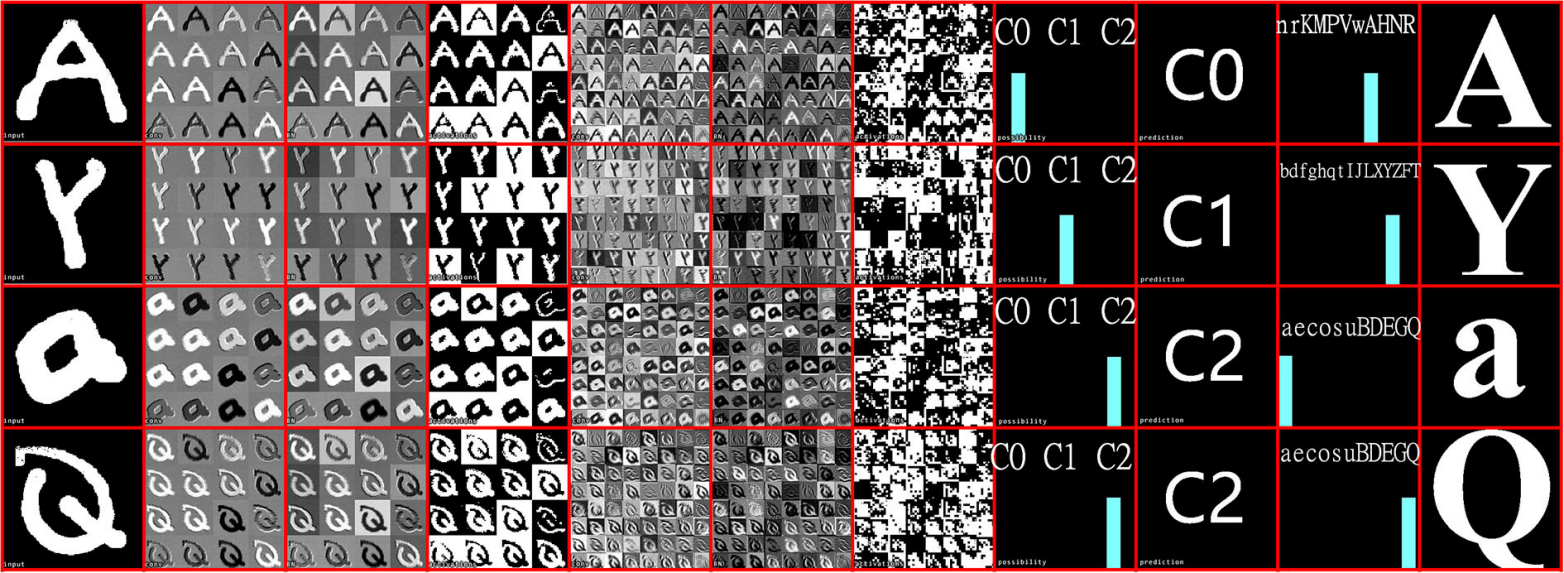
Examples of CNN tree inference on SCAMP-5d. For each letter from left to right: (1) Real-time hand-written input image by facing the SCAMP to a writing pad. (2) first convolutional layer. (3) convolutional layer after batch norm. (4) binary feature maps after maxpooling and activation function. (5) second convolutional layer. (6) convolutional layer after batch norm. (7) binary feature maps for fully-connected layer after maxpooling and activation function. (8) prediction bar for first CNN inference results (Category 0, 1, 2). (9) Switch CNN inference results. (10) prediction bar for the second CNN inference results. (11) final CNN inference results. Notice that for each letter inference process, visualization is only for one CNN.

To evaluate the CNN implementation on SCAMP, Supplementary Figure S3 compares the first fully-connected neuron values between SCAMP and Simulation on PC because noises are mainly caused by analog signal processing on the PPA and there would be no noise introduced to the last fully-connected layer afterward since it is implemented on the micro-controller. As shown in Supplementary Figure S3, the neuron values from SCAMP using digital summation are close to the ground truth in simulation and the average absolute error measured is around 22 for each neuron with a value ranging from 0 to 500. Figure 12 visualizes the on-sensor inference process from the input image, convolutions, activations, and final predictions. The final measured accuracy with EMNIST datasets on the PPA is around 82%, which sees a 4–5% accuracy gap from the groundtruth in the PC simulation. Supplementary Figure S9 demonstrates some of the live demos of letter classification. In addition, a break down of time cost within one branch of networks for each inference step can be seen from Table 2.

**Table 2:** Computation time breakdown CNN of a single branch.

**Processing steps**	**Approximately time cost (*μs*)**
Imaging and thresholding	35
Character resize and duplication	359
1st Image convolution	184
1st Batch norm and activation	235
2nd Group convolution	184
4×4 maxpooling	34
2nd Batch norm and activation	235
1st fully-connected layer	4,318
2nd fully-connected layer	11
Total time cost	5,595 (178 FPS)
number of weights	≈133k
model size	≈0.127 KB

### 6.2. FCN inference, experiments, and evaluation

**Figure 13:**
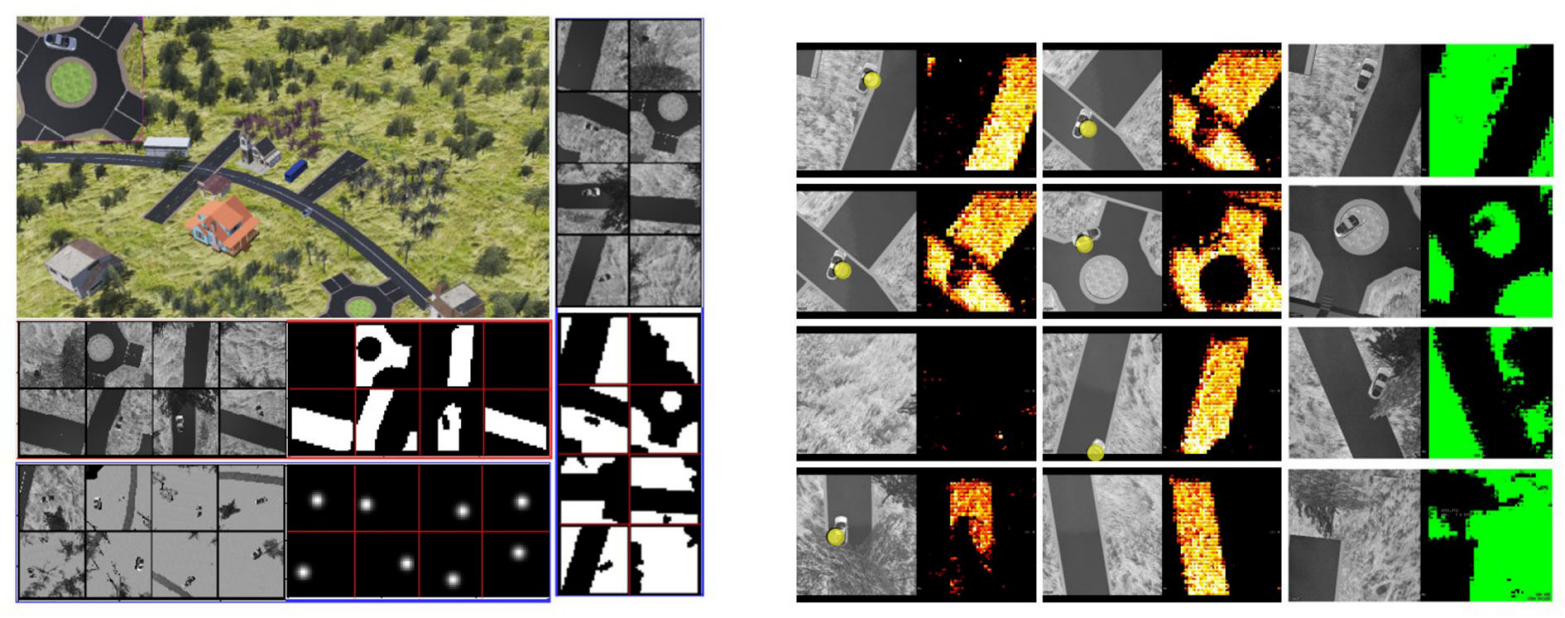
Data collection environment and inference results. Left) The data collection environment where a vehicle is moving around and images are generated from a bird's eye view of a simulated drone; top left: A robot simulator for environment setup and collection of training data; middle left: The collected images from the drone's camera are converted into gray-scale images for the real PPA and the segmentation annotations of the road. Right) The training and annotated datasets for grass segmentation; bottom left: this study uses the Gaussian distribution to represent the vehicle position within an image; right: FCN inference results on-sensor. The left column is the input gray-scale image on the sensor with yellow dots indicating the FCN inference localization prediction and the right column is the inference results for coarse segmentation on the sensor. The density and distribution of colorful points (right) represent the possibility of the position of the road (bright yellow) and grass (green) segmentation. The experimental performance for localization (accuracy) and segmentation (IoU) on the PPA can be seen in Figure 14. An example video can be seen at https://youtu.be/Z_ydv_0DRnM.

This section demonstrates the application of the proposed network architecture to coarse segmentation and object 2D localization from a bird's eye view. We implement the FCN algorithm on the SCAMP vision system hardware (Supplementary Figure S8). We created an environment in the Webots^2^ (Michel, 2004) robot simulator (Figure 13, Left) for data collection and the validation of FCN deployment on the sensor. Training, testing, and validation datasets are collected by repeatedly taking images from a flying drone equipped with a simulated ‘SCAMP’ and then validation images are sent to the real PPA hardware for inference. Binarized FCN is trained offline based on these datasets with the method proposed in Section 3. The whole neural network for both coarse segmentation and localization is performed on the sensor.

**Figure 14:**
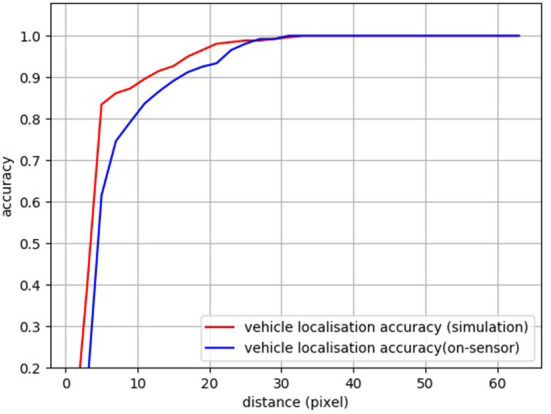
Performance comparison between simulation and on the sensor for localization task.

Figure 13, Left shows the samples of collected datasets and their annotations for the segmentation of road and grass. To validate the performance of the proposed network on different tasks, a road and grass coarse segmentation is explored in this section. As shown in Figure 13, Left, we directly use the road/grass shape as the ground truth for coarse segmentation. Notice that the trees and grass areas often share similar gray-scale levels with the road, making coarse segmentation unfeasible by simply using binary thresholding. **Table 4** shows the Intersection over Union (IoU) performance comparison between FCN inference on simulation and sensor. Specifically, IoU is measured here by counting the number of intersected pixels over the number of united pixels of the predictions and groundtruth. Some of the results can be seen in Figure 13. Table 3 compares the experimental results on the sensor and its counterpart baseline on the computer with identical neural networks and validation images. More results can be seen from the Supplementary Video.

**Table 3:** Intersection over union (IoU) performance comparison between simulation and on the sensor for coarse segmentation.

**Task**	**IoU**
Road segmentation on simulation	74.0%
Road segmentation on sensor	69.3%
Grass segmentation on simulation	76.6%
Grass segmentation on sensor	72.9%

#### 6.2.1. Object detection

We also implemented an object detection task, based on the heat map. As for the object localization, rather than using the vehicle segmentation image as the ground truth for training, we use Gaussian position distribution (Figure 13, Left) as the ground truth since the probability distribution is adequate to represent the object's 2D localization. For the validation, a distance threshold is set from 0 to 63 to count the number of predictions with a distance to the groundtruth that falls into this threshold. A zero distance means a perfect prediction. The final localization is obtained by the weighted sum of all the possible positions. After the test, within a distance of 10 pixels, the vehicle localization accuracy for simulation and SCAMP is around 88 and 83%, respectively (Figure 13). Table 4 shows the FCN performance in terms of time, power consumption, and model size.

**Table 4:** Computation time, performance, and weights for heat map generation with the binarized fully convolutional network (FCN) on sensor.

**Processing steps**	**Time cost (*μs*)**
Image replication	112
1st Image convolution	184
1st Batch norm and activation	235
kernel filter activation and replication	212
2nd Group convolution	184×4 = 736
2×2 maxpooling	35×4 = 140
2nd Batch norm and activation	235×4 = 940
Third convolutional layer	966
Total time cost	3,525 (283 FPS)
number of weights	2,578
power consumption	≈1.5 W
model size	≈0.31 KB

Notice that there is about a 5–6% performance gap for the experiment on sensor compared to the simulation. This is due to noise in the convolution operation performed on AREG because of the inherent non-idealities of analog computation (Carey et al., 2014) and some random bit-flipping errors observed in DREG when performing massively parallel shifting and replications. Mitigation of these issues requires further software or hardware solutions but is mostly due to the prototype nature of the SCAMP hardware. In this study, we aimed to strike a balance between network complexity and viability for deployment upon the available PPA prototype hardware. Pixel-wise accurate segmentation, with a quality equal to one that can be obtained using a CPU/GPUs hardware, using an embedded low-power SCAMP-5d vision system, is still a challenging task with current hardware and neural network architecture.

#### 6.2.2. Shared convolutional layer for multiple tasks with FCN

**Figure 15:**
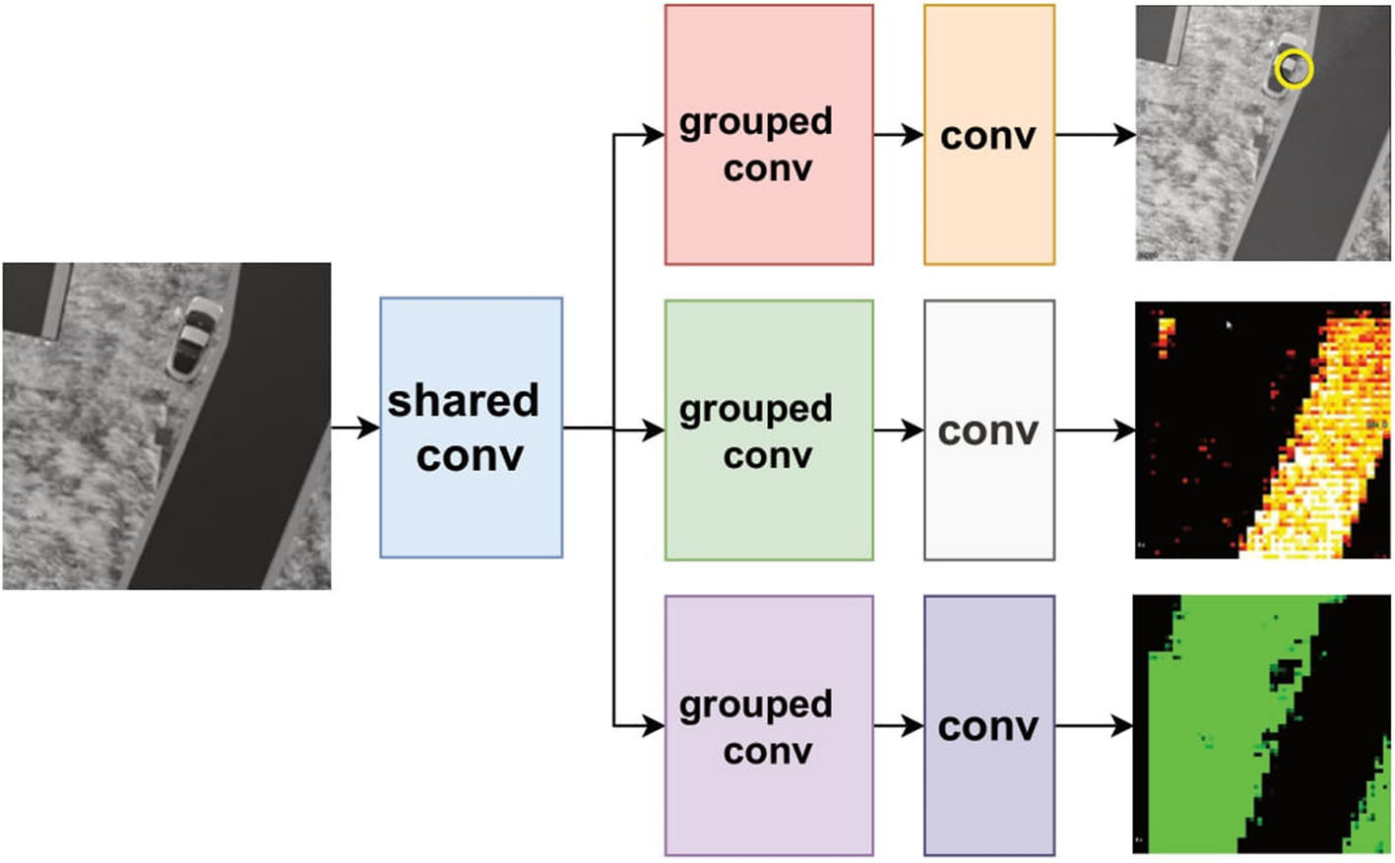
An FCN tree architecture with a shared convolutional layer for three task-specific FCNs. One of the task-specific FCNs performs object detection (yellow circle), and two of the region segmentation (road and grass).

We use a dynamic model swapping strategy to run these three networks on the PPA by sharing the first convolutional layer among these networks (shown in Figure 15) considering the same testing environment and similar network structure for these three networks. With this shared weights scheme, less storage requirement for DREG and higher inference efficiency without uploading extra weights from the flash memory can be obtained for three networks. Rather than train these three networks concurrently which might cause an unbalanced training process for each task because of their different scale of loss (Sener and Koltun, 2018), we adopt a straightforward but efficient method by training the neural network for localization first, locking the weights in the first convolutional layer and then training the other segmentation networks, respectively. The reason to train these three networks in this way is that the localization task is more sensitive to the final heat map prediction hence better to train separately, while segmentation for road and grass are similar tasks that are more tolerant to the final heat map prediction. Some selected results can be seen in Figure 15. A break down of time cost within one branch of networks for each inference step can be seen in Table 2. Notice that, according to our test, it takes around 26 ms to upload a CNN model from the external flash memory into a DREG. However, this issue can be solved by pre-storing the binary models into DREGs or AREGs before the CNN inference process.

## 7. Conclusion

In this study, we consider methods to improve the efficient embedding and deployment of neural networks on resource-constrained Pixel processor Arrays (PPAs). We propose a series of methods including purely binarized networks, group convolution, fully-connected layer with the digital summation, and network tree with dynamic swapping. We demonstrate performance on classification, localization, and segmentation tasks. By integrating these techniques, a deeper neural network with better inference capacity is enabled on the SCAMP PPA, hence making more sophisticated tasks possible. In contrast to previous studies that have mainly focused on classification with fully-connected layers, for the first time, we exploit the on-sensor FCN architecture with novel implementation methods. We validate, using the SCAMP-5 PPA, the visual competencies of region segmentation and target object localization with a latency of 3.5 ms for each inference. In addition, this study explores a new CNN tree architecture by running several neural networks in sequence according to the previous network output. Each network in the tree fully takes advantage of the hardware resources of the embedded device. With this method deeper and wider CNN/FCN tree can be deployed upon SCAMP and other embedded devices. Two experiments of 37 letter classification with a network tree architecture and object coarse segmentation with a shared convolution layer demonstrate the effectiveness of the proposed binarized CNN and implementation method on the SCAMP vision system. Some classic and deeper neural networks (such as AlexNet and VGG-16) are still challenging to implement without further hardware development progress of PPAs. We believe there is still a range of important developments that can be made for PPAs, which together with further advances in hardware will help improve lag, energy consumption, and data bandwidth for embedded vision systems.

## Data availability statement

The raw data supporting the conclusions of this article will be made available by the authors, without undue reservation.

## Author contributions

YL proposed the binarized neural networks on the PPA with their implementations and experimental validation. LB contributed to the accurate summation methods based on the DREGs. RF contributed to the idea of alphabet classification and its categorization methods. PD and WM-C planned and supervised the project. WM-C proposed the idea of model dynamic swapping. WM-C, PD, and RF reviewed the results and the final version of the manuscript. All authors contributed to the article and approved the submitted version.

## Funding

This work was supported by National Key R&D Program of China (Grant No. 2020AAA0108100), UK EPSRC EP/M019454/1, EPSRC Centre for Doctoral Training in Future Autonomous and Robotic Systems: FARSCOPE, and China Scholarship Council (CSC, No. 201700260083).

## Conflict of interest

Author LB is currently employed by Pixelcore Research. Author PD was employed by Pixelcore Research. Author WM-C was employed by Amazon.com. The remaining authors declare that the research was conducted in the absence of any commercial or financial relationships that could be construed as a potential conflict of interest.

## Publisher's note

All claims expressed in this article are solely those of the authors and do not necessarily represent those of their affiliated organizations, or those of the publisher, the editors and the reviewers. Any product that may be evaluated in this article, or claim that may be made by its manufacturer, is not guaranteed or endorsed by the publisher.

## Footnotes

^1^ https://youtu.be/a2VO3aWHnYc

^2^ https://cyberbotics.com/
