# Supplementary material for: On-sensor binarized CNN inference with dynamic model swapping in pixel processor arrays
Source: Front Neurosci. 2022 Aug 15;16:909448. doi: 10.3389/fnins.2022.909448 (PMC9421154; doi:10.3389/fnins.2022.909448)
Supplement: Supplementary file 1 [file Data_Sheet_1.pdf]

frontiers

Supplementary Material

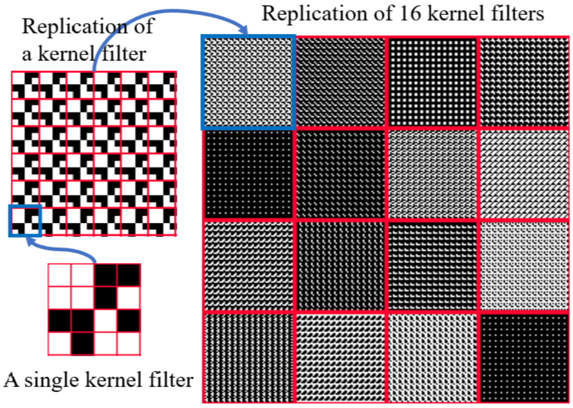

Figure S1. The layout of 16 binarized convolutional kernels in a DREG.

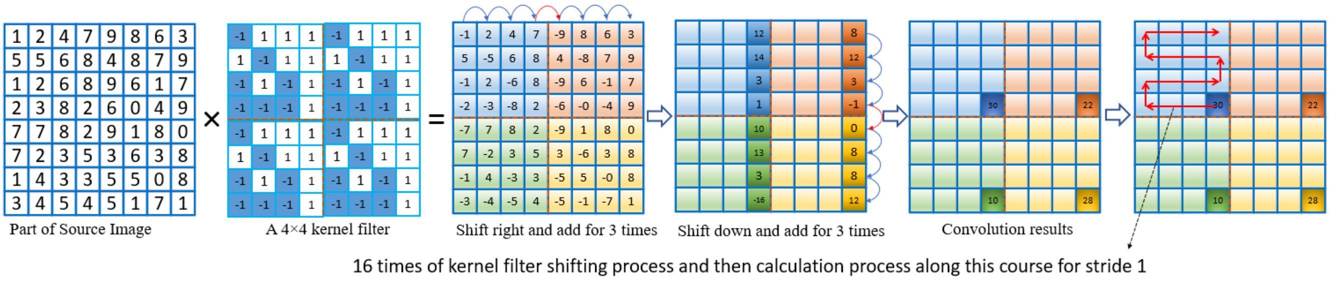

Figure S2. Image convolution on PPA with sign inversion, bit-shifting, and addition.

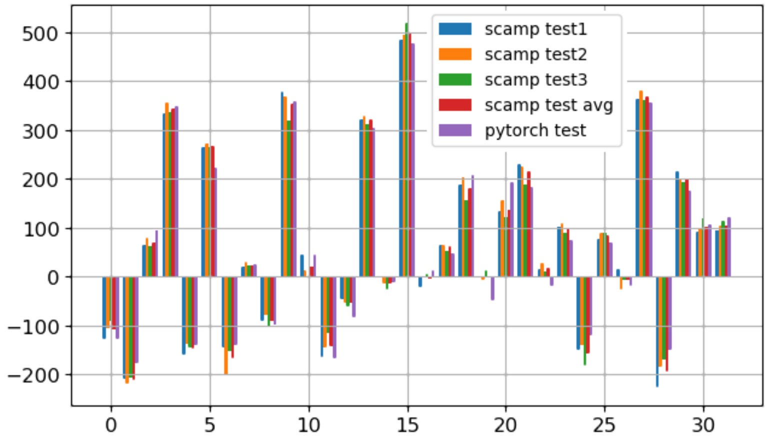

Figure S3. CNN neuron values (32 neurons in the first fully-connected layer) comparison between PPA and PC simulation for the first fully-connected layer. A single image is tested three times on SCAMP and neuron values and their average values are recorded to compare with groundtruth values from PC.

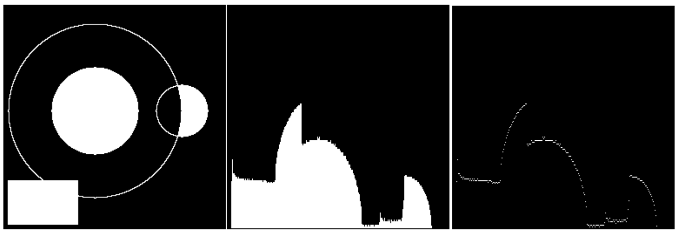

Figure S4. Stages of the 'sandcastle summation' method used for bit counting. From left to right, example of DREG content to be summed, the stacked 'sandcastle' of set pixels formed, the pixels along the top of the 'sandcastle' whose extracted locations are used to calculate the total number of set pixels.

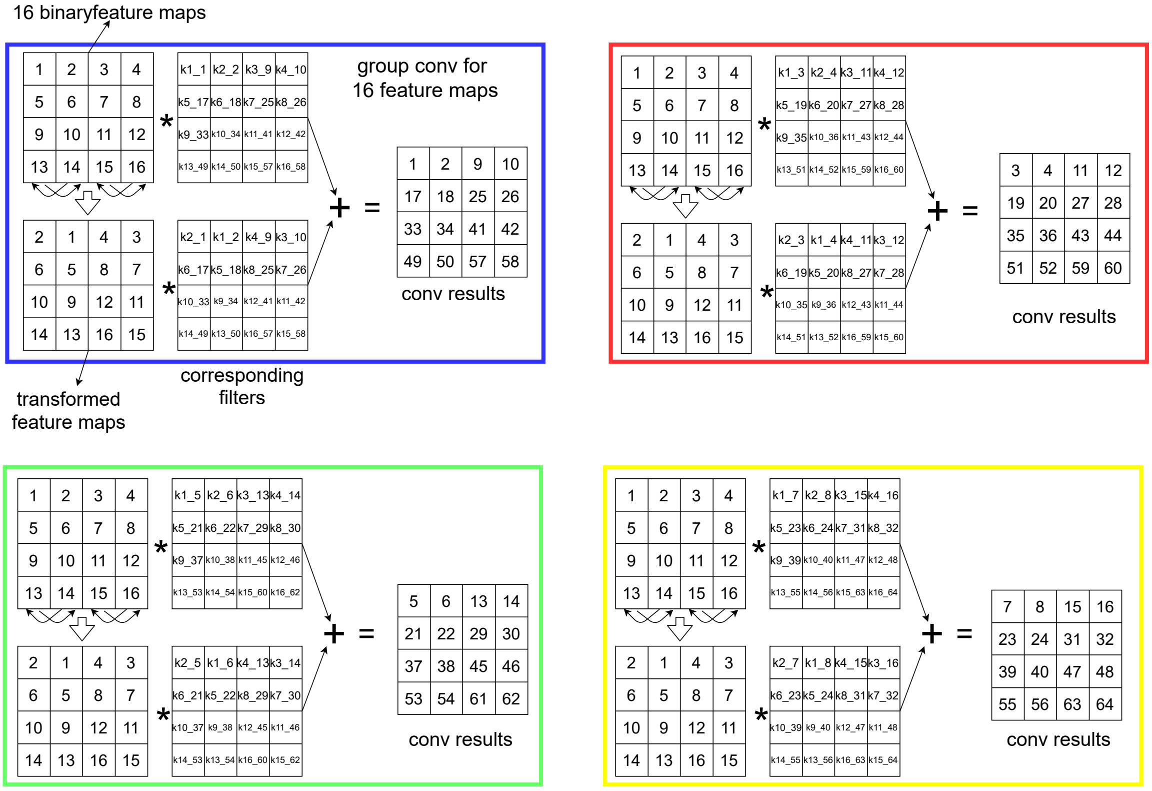

Figure S5. 64 feature map generation on sensor after group convolution.

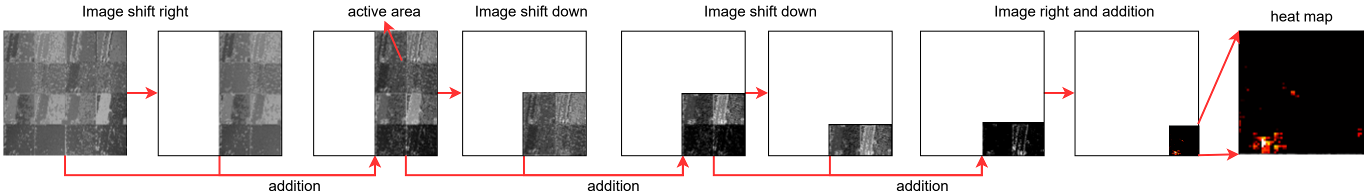

Figure S6. Feature map shifting and addition process: the final heat map is generated by adding the feature maps from the third layer.

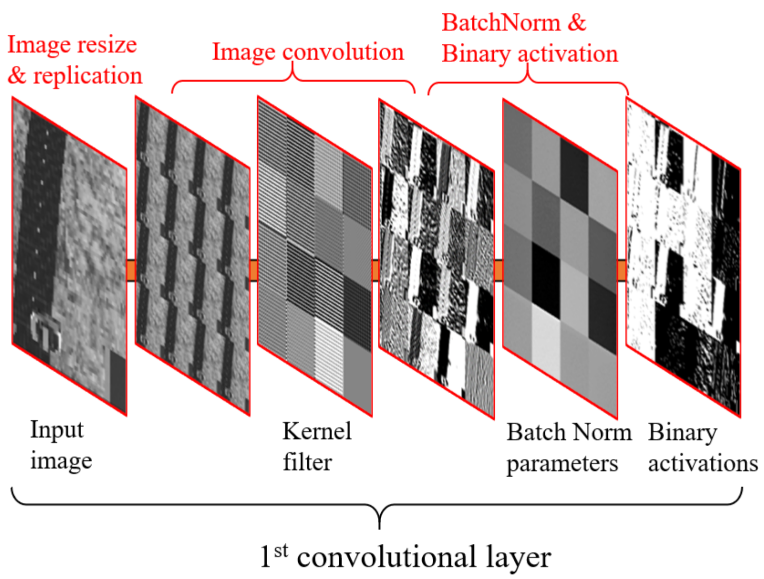

Figure S7. First convolutional layer of the FCN.

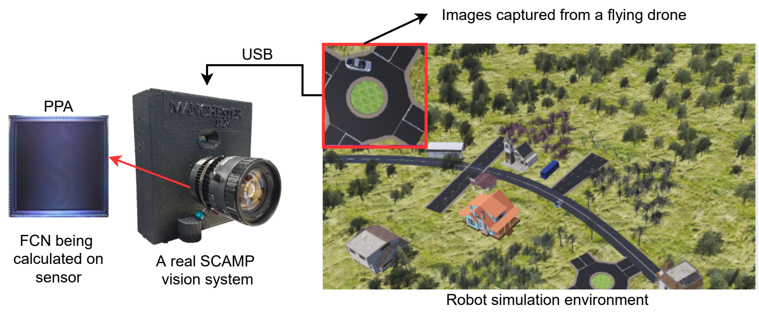

Figure S8. Experimental setups using a robot simulator and a real SCAMP vision system where the neural networks are fully computed on the PPA.

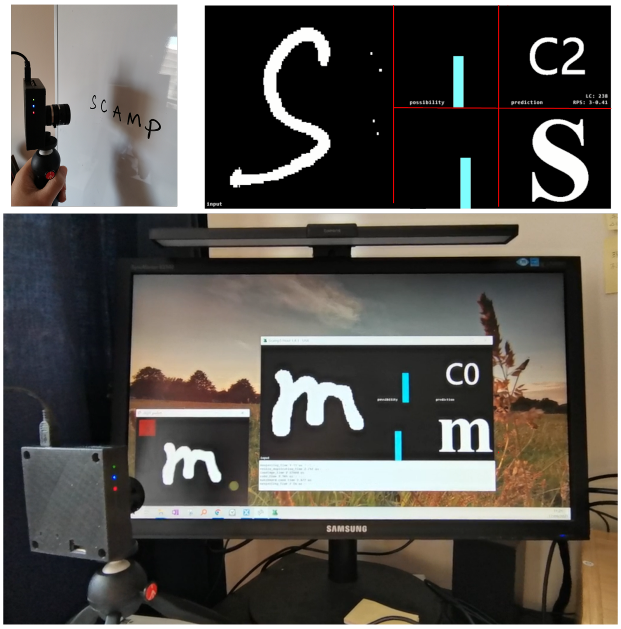

Figure S9. A live demo by facing SCAMP to handwritten letter on a whiteboard and screen drawing pad. Top left, facing the SCAMP to a whiteboard and the inference results on the top right. Bottom: facing the SCAMP to a hand-written letter on a drawing pad (bottom left). More details can be seen from the experimental video `https://youtu.be/8V9vXhXw8X8`

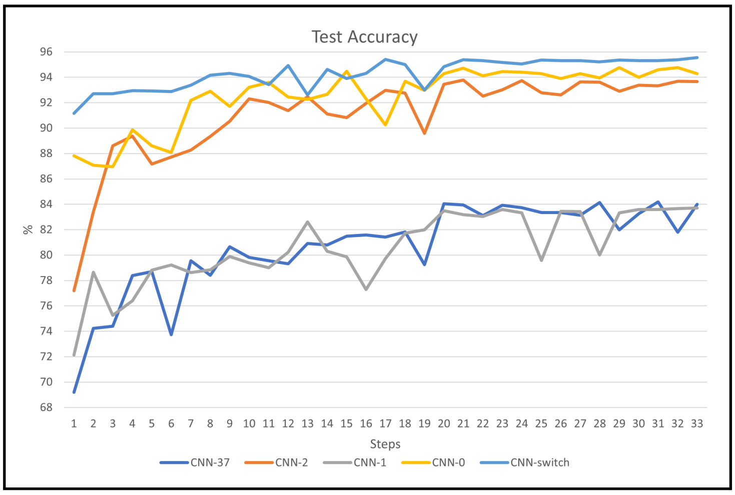

Figure S10. CNN tree training performance. We compared the CNN tree with a single CNN scheme in terms of accuracy. With the same binarized CNN architecture of 2 convolution and 2 fully connected layers, the overall accuracy of CNN tree is better than single CNN. Although CNN tree sacrifices the efficiency by running multiple CNNs sequentially, it provides a solution to combine several networks for a comparatively complicated task.
